# EGFR-Targeted Extracellular Vesicles Potentiate Doxorubicin-Induced Apoptosis and Tumor Suppression in Colorectal Cancer

**DOI:** 10.3390/ijms27083693

**Published:** 2026-04-21

**Authors:** Chan Mi Lee, Ji Won Choi, Do Sang Lee, Joo Won Moon, Jin Beom Cho, Jung Hoon Bae

**Affiliations:** 1 Central Institute of Surgery, Seoul St. Mary’s Hospital, College of Medicine, The Catholic University of Korea, Seoul 06591, Republic of Korea; cksal7873@cmcnu.or.kr (C.M.L.); qrt97@cmcnu.or.kr (J.W.C.); dosangs@catholic.ac.kr (D.S.L.); mey8010@cmcnu.or.kr (J.W.M.); jinbum21@catholic.ac.kr (J.B.C.); 2 Department of Surgery, Seoul St. Mary’s Hospital, College of Medicine, The Catholic University of Korea, Seoul 06591, Republic of Korea; 3 Division of Colorectal and Anorectal Surgery, Department of Surgery, Uijeongbu St. Mary’s Hospital, College of Medicine, The Catholic University of Korea, Uijeongbu 11765, Republic of Korea

**Keywords:** colorectal cancer (CRC), extracellular vesicles (EVs), GE11, EGFR Target EV, p53-mediated apoptosis

## Abstract

Colorectal cancer (CRC), characterized by epidermal growth factor receptor (EGFR) overexpression, is often associated with poor prognosis and limited therapeutic response to conventional chemotherapy. In this study, we developed EGFR-targeted extracellular vesicles (EGFR-tEVs) by transiently engineering donor cells to display the GE11 peptide, aiming to enhance the precision of doxorubicin (Dox) delivery. The physicochemical properties of EGFR-tEVs were characterized using TEM, NTA, and Western blot. In vitro, EGFR-tEV-Dox exhibited increased cellular uptake in EGFR-overexpressing HCT-116 cells, leading to the activation of the p53-Bax-cleaved PARP1 apoptotic pathway. Notably, while Dox treatment induced p53 in normal colon fibroblasts (CCD18-Co), it did not trigger significant Bax activation or PARP1 cleavage, suggesting a preference for survival-related signaling in non-malignant cells. In a xenograft mouse model, EGFR-tEVs + Dox administration resulted in a 33.1% reduction in tumor volume and an 82.8% decrease in Ki-67 expression compared to the control group. These results indicate that transient receptor-mediated targeting enhances functional drug delivery to malignant tissues while minimizing pro-apoptotic induction in normal cells. Our findings suggest that EGFR-tEVs + Dox represents a balanced therapeutic strategy that improves antitumor efficacy with a favorable safety profile for EGFR-positive colorectal cancer.

## 1. Introduction

Colorectal cancer (CRC) remains the third most frequently diagnosed cancer globally and the second leading cause of cancer-related mortality, accounting for approximately 1.9 million new cases and over 900,000 deaths annually [1,2,3]. Despite advances in screening, the global burden of CRC is projected to increase substantially by 2050 due to rising incidence in younger populations [4,5]. Currently, surgical resection and systemic chemotherapy are the primary treatment modalities; however, the prognosis for patients with advanced metastatic CRC (mCRC) remains dismal, with a 5-year survival rate still hovering below 15% [5,6]. The limited efficacy of conventional surgery is often attributed to late-stage diagnosis and high recurrence rates, while systemic chemotherapy is frequently hampered by dose-limiting toxicities and the lack of tumor specificity, which induces severe adverse effects in normal tissues [7]. To address these limitations, targeted therapies have emerged as a promising approach that improves treatment by selectively targeting cancer cells and potentially extending patient survival [8,9].

To overcome the limitations of conventional treatments, we used cell-derived extracellular vesicles (EVs) as drug delivery vehicles. EVs play a vital role in mediating intercellular communication by transporting membrane proteins, lipids, and nucleic acids [10]. EVs, in particular, are small vesicles that mediate communication by transferring specific proteins, lipids, miRNAs, and mRNAs, thereby influencing target cells. These properties of EVs make them crucial in disease treatment and diagnosis [11,12]. EVs express specific surface markers reflective of their parent cells. These surface proteins facilitate their binding to specific target cells, thus enabling targeted delivery [13,14,15]. Hence, by utilizing receptors or surface markers that enable selective binding, EVs offer potential for targeted drug delivery to specific cells. This strategy allows for precise drug delivery to the desired location while minimizing side effects.

Abnormal expression levels of the epidermal growth factor receptor (EGFR) and human epidermal growth factor receptor 2 (HER2) have been observed in various cancer types, including CRC. Specifically, EGFR is overexpressed in 60–80% of advanced CRC cases, and elevated EGFR levels are correlated with poorer patient survival outcomes [16,17,18]. FDA has approved several EGFR-targeted therapies, including tyrosine kinase inhibitors (TKIs) and monoclonal antibodies, which are widely used as critical treatment options for non-small cell lung cancer (NSCLC), CRC, head and neck cancers, and other malignancies [19]. However, the clinical efficacy of these agents is frequently hampered by the development of secondary mutations or the activation of alternative signaling pathways, and systemic administration of antibodies can still lead to off-target skin and gastrointestinal toxicities [20,21,22]. Therefore, there is an urgent unmet need for a delivery platform that can encapsulate potent chemotherapeutic agents and deliver them specifically to EGFR-overexpressing tumor tissues, thereby enhancing local drug concentration while sparing healthy cells. Notably, the GE11 peptide (a 12-amino acid sequence) binds to EGFR with high affinity and enables selective targeting of cancer cells without activating EGFR signaling [23,24].

Based on these studies, we explored the potential of EVs as a drug delivery system. We hypothesized that EVs engineered to specifically target EGFR on cancer cells could effectively deliver therapeutic agents. This strategy aims to enhance the therapeutic efficacy while selectively targeting malignant cells. By employing EVs for the targeted delivery of agents that block EGFR signaling, it is possible to inhibit the aberrant EGFR activation and suppress tumor growth. Our findings demonstrate that EGFR-targeted EVs carrying doxorubicin (EGFR-tExo + Dox) significantly outperform conventional Dox at the equivalent dosage in treating HCT-116 cells, indicating an improved therapeutic efficiency. Furthermore, we evaluated the safety profile of this system using CCD-18co, a normal colon fibroblast cell line, to demonstrate that EGFR-tEVs can minimize off-target effects and pro-apoptotic induction in non-malignant tissues, thereby offering a more balanced and safer therapeutic approach compared to free doxorubicin.

## 2. Results

### 2.1. Identification and Characterization of the EVs

**Figure 1:**
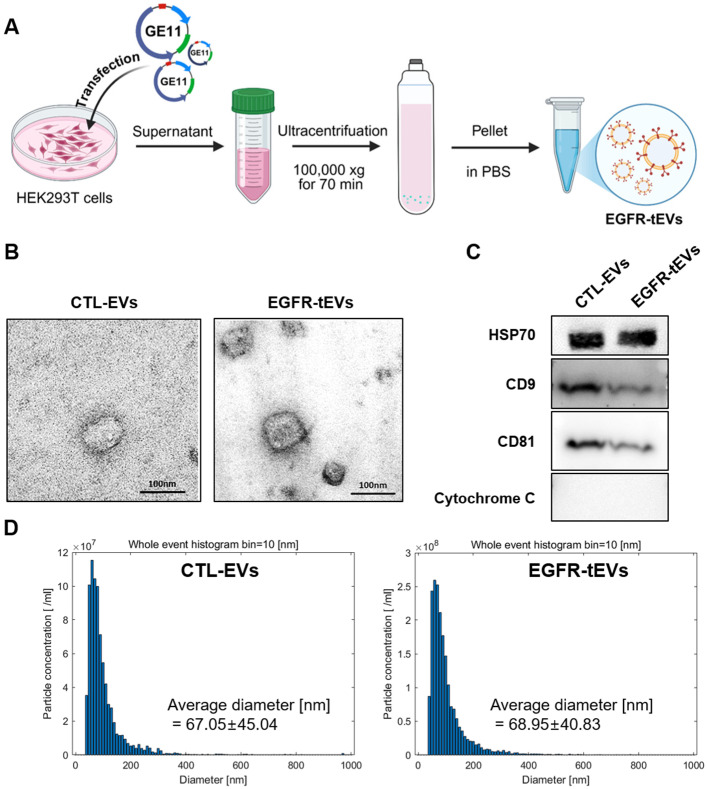
Isolation and characterization of extracellular vesicles (EVs). (**A**) Schematic illustration of EV isolation from HEK293T cells following transfection with the GE11 peptide for EGFR targeting. (**B**) Transmission electron microscopy (TEM) images of control EVs (CTL-EVs) and EGFR-targeted EVs (EGFR-tEVs). Vesicles displayed a typical round or cup-shaped morphology with diameters ranging from 30 to 150 nm. Scale bar = 100 nm. (**C**) Western blot analysis of EV-associated proteins. Equal amounts of total protein from EV preparations were loaded. Canonical EV markers CD9, CD81, and HSP70 were detected in both CTL-EVs and EGFR-tEVs. The intracellular protein cytochrome c was not detected in EV samples, suggesting minimal contamination with intracellular components. (**D**) Nanoparticle tracking analysis (NTA) showing particle size distribution. The mean particle diameter was 67.05 ± 45.04 nm for CTL-EVs and 68.95 ± 40.83 nm for EGFR-tEVs. Data are presented as mean ± SD from n = 3 independent EV isolations.

Control-extracellular vesicles (CTL-EVs) were isolated from HEK293T cells by differential centrifugation without transfection, while EGFR-targeted extracellular vesicles (EGFR-tEVs) were obtained after transfection with the GE11 vector (Figure 1A). The EVs were characterized using TEM, Western blot, and NTA. TEM and NTA analyses revealed that both CTL-EVs and EGFR-tEVs exhibited typical cup-shaped morphology and a size distribution of 30–150 nm, with average diameters of 67.05 ± 45.04 nm and 68.95 ± 40.83 nm, respectively (Figure 1B,D). The average diameters were 67.05 ± 45.04 nm for CTL-EVs and 68.95 ± 40.83 nm for EGFR-tEVs (Figure 1B,D). Western blot analysis confirmed the presence of canonical EV markers CD9, CD81, and HSP70 in both CTL-EVs and EGFR-tEVs. Cytochrome c, used as a negative marker for intracellular contamination, was not detected in either EV preparation (Figure 1C), suggesting minimal contamination with intracellular proteins. These results support the successful isolation of CTL-EVs and EGFR-tEVs with typical EV characteristics.

### 2.2. Enhanced Cellular Uptake of EGFR-tEVs in HCT-116 Cells

**Figure 2:**
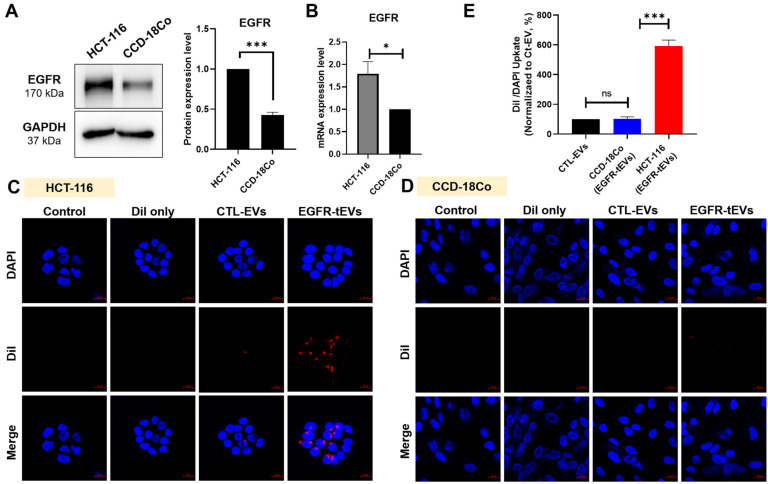
Enhanced uptake of EGFR-targeted EVs in EGFR-expressing colorectal cancer cells. (**A**) EGFR protein expression in HCT-116 colorectal cancer cells and normal cell lines (CCD-18Co and CCD-986sk) was analyzed by Western blot. HCT-116 cells exhibited higher EGFR expression compared with normal cells. (**B**) Relative EGFR mRNA expression was quantified by qRT-PCR. Data are normalized to internal controls and presented relative to CCD-986sk cells. (**C**) Representative confocal microscopy images showing uptake of Dil-labeled CTL-EVs and EGFR-tEVs in HCT-116 cells after 24 h incubation. Scale bar = 20 μm. (**D**) Representative confocal microscopy images showing uptake of Dil-labeled CTL-EVs and EGFR-tEVs in CCD-18Co cells after 24 h incubation. Scale bar = 20 μm. (**E**) Quantitative analysis of EVs uptake normalized to DAPI-positive nuclei. Data are shown as mean ± SD from n = 3 independent experiments. * *p* < 0.05, *** *p* < 0.001, and ns, not significant.

The protein and mRNA expression levels of EGFR were analyzed in the colorectal cancer cell line HCT-116 and the normal colon cell line CCD-18Co. EGFR expression was significantly higher in HCT-116 cells compared with the normal cell lines, consistent with previous reports indicating elevated EGFR expression in cancer cells (Figure 2A,B). To evaluate the effect of EGFR targeting on EV uptake, Dil-labeled CTL-EVs and EGFR-tEVs were incubated with HCT-116 cells for 24 h and examined by confocal microscopy. Fluorescence imaging revealed markedly increased red fluorescent signals in the cytoplasm of cells treated with EGFR-tEVs compared to those treated with CTL-EVs (Figure 2C). No significant fluorescence signal was observed in the Dil-only control group. Quantitative analysis of fluorescence intensity per cell was performed using ImageJ software (version 1.54g). After normalization to the number of DAPI-stained nuclei, the uptake of CTL-EVs was set to 100%, and the relative uptake of EGFR-tEVs in HCT-116 cells was calculated as 592.2 ± 32.6%, demonstrating a statistically significant increase (Figure 2E). In contrast, uptake experiments in CCD-18Co cells, which exhibit low EGFR expression, showed no significant difference in fluorescence intensity between CTL-EVs and EGFR-tEVs (Figure 2D,E). These results suggest that EGFR targeting effectively and selectively enhances EV internalization in EGFR-overexpressing colorectal cancer cells, while minimizing non-specific uptake in normal cells.

### 2.3. EGFR-tEVs Enhance Doxorubicin-Mediated Apoptosis and Suppress Cell Migration in HCT-116 Cells

**Figure 3:**
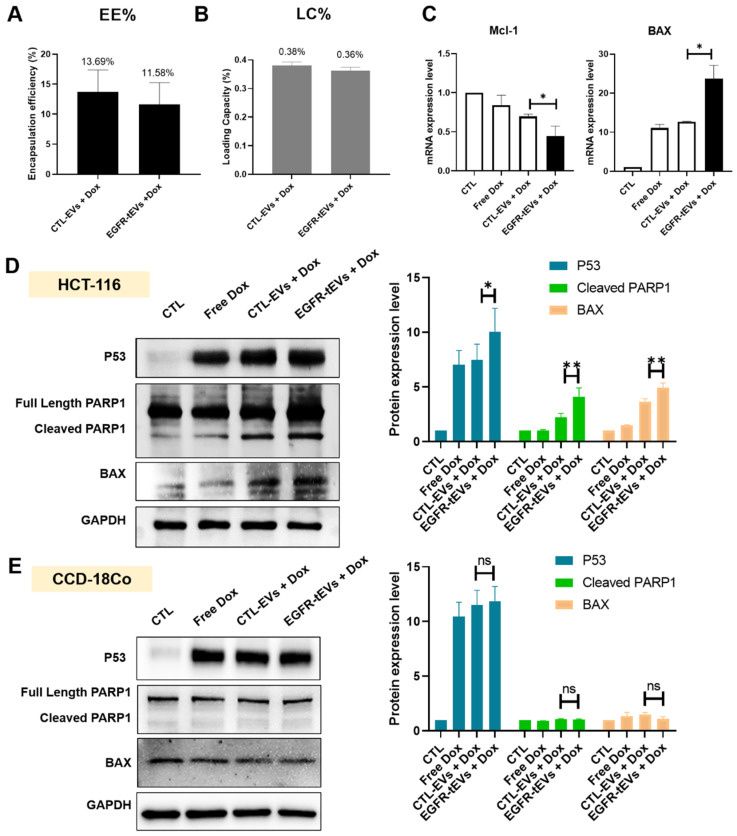
EGFR-tEVs selectively enhance doxorubicin-induced apoptosis in HCT-116 cells. (**A**,**B**) Characterization of doxorubicin loading into EVs. (**A**) Encapsulation efficiency (EE%) and (**B**) Loading capacity (LC%) of doxorubicin in CTL-EVs and EGFR-tEVs after incubation with 2 μM doxorubicin. Data are presented as mean ± SD (n = 3). (**C**,**D**) Pro-apoptotic effects of EGFR-tEVs + Dox in HCT-116 cells. Cells were treated for 24 h with free Dox, CTL-EVs + Dox, or EGFR-tEVs + Dox. (**C**) Relative mRNA expression levels of BAX and *Mcl-1* quantified by RT-qPCR. (**D**) Protein expression levels of p53, cleaved PARP1, and BAX were analyzed by Western blot. Densitometric quantification of protein bands normalized to GAPDH. (**E**) Selective safety profile in CCD-18Co cells. Protein expression of p53, BAX, and cleaved PARP1 in normal human colon cells (CCD-18Co) following 24 h treatment. Note the lack of significant difference between the free Dox and EGFR-tEVs + Dox groups, indicating reduced off-target toxicity. Data are presented as mean ± SD from n = 3 independent experiments. * *p* < 0.05, ** *p* < 0.01, and ns, not significant.

Prior to evaluating therapeutic efficacy, the drug loading parameters were characterized. The encapsulation efficiency (EE%) of doxorubicin was 13.69% for CTL-EVs and 11.58% for EGFR-tEVs, with corresponding loading capacities (LC%) of 0.38% and 0.36%, respectively (Figure 3A,B). Based on these parameters, we investigated the apoptotic impact of the delivered doxorubicin. In HCT-116 cells, treatment with EGFR-tEVs loaded with doxorubicin (EGFR-tEVs + Dox) significantly increased the expression of p53, cleaved PARP1, and BAX compared with CTL-EVs + Dox or free Dox, indicating enhanced activation of the intrinsic apoptotic pathway (Figure 3D). This was further supported by qRT-PCR analysis, which showed a significant upregulation of BAX mRNA and downregulation of *Mcl-1* mRNA in the EGFR-tEVs + Dox group (Figure 3C). Notably, to evaluate the cancer-specific targeting and safety of EGFR-tEVs, we examined their effects on CCD-18Co cells, a normal human colon cell line. In contrast to the results in HCT-116 cells, the expression levels of p53, BAX, and cleaved PARP1 in CCD-18Co cells showed no significant differences between the free Dox and EGFR-tEVs + Dox groups (Figure 3E). Collective findings from both protein expression and wound healing assays demonstrate that EGFR-tEVs + Dox not only potentiate apoptotic signaling but also effectively suppress the migratory capacity of HCT-116 cells, highlighting their potential as a precise therapeutic delivery platform.

### 2.4. EGFR-tEVs Enhance Tumor-Targeting Ability in a Xenograft Mouse Model

**Figure 4:**
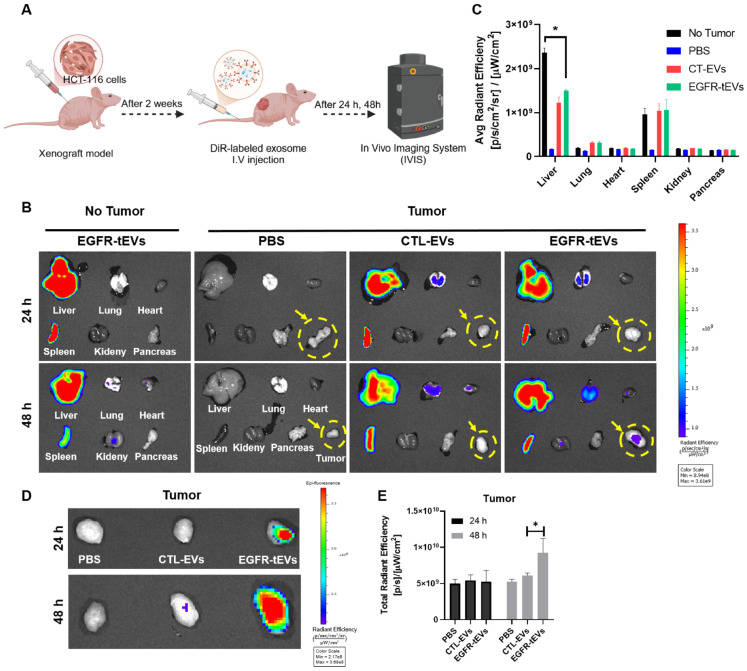
In vivo tumor-targeting ability of EGFR-tEVs in an HCT-116 xenograft model. (**A**) Experimental design for evaluating biodistribution of DiR-labeled EGFR-tEVs in tumor-bearing and tumor-free mice. (**B**) Representative ex vivo fluorescence images of major organs (liver, lung, heart, spleen, kidney, pancreas) and tumor tissues collected 24 h after intravenous injection of DiR-labeled EGFR-tEVs (1 × 10^8^ particles). Yellow dotted circles indicate the location of the tumor tissues. (**C**) Quantification of fluorescence intensity in each organ and tumor tissue. (**D**) Representative fluorescence images highlighting tumor accumulation in tumor-bearing mice. (**E**) Quantitative comparison of fluorescence intensity in tumor tissues between groups. The color scale represents the radiant efficiency in (p/sec/cm^2^/sr)/(μW/cm^2^). Data are presented as mean ± SD (n = 3 mice per group). Statistical analysis was performed using Student’s *t*-test. * *p* < 0.05.

To evaluate the in vivo tumor-targeting and tissue-homing capability of EGFR-tEVs, an HCT-116 cell-based xenograft mouse model was established. EGFR-tEVs were labeled with DiR, a fluorescent dye that strongly associates with the EV membrane, and intravenously administered to both tumor-bearing and non-tumor control mice. In vivo fluorescence imaging was performed using IVIS (Figure 4A). At 24 and 48 h post-injection, major organs (liver, lung, heart, spleen, kidneys, and pancreas) and tumor tissues were collected for ex vivo imaging. EVs exhibited the strongest fluorescent signals in the liver, likely due to clearance by the reticuloendothelial system (RES) [25]. In contrast, significant accumulation of EGFR-tEVs was observed in the tumor tissues of tumor-bearing mice (Figure 4B). Quantitative analysis of fluorescence intensity revealed that EGFR-tEV accumulation in the liver of tumor-bearing mice was significantly lower than that in non-tumor controls, suggesting that a portion of EVs trafficked to the tumor, thereby reducing their hepatic accumulation (Figure 4C). Among all groups, the highest fluorescent signals were detected in the tumors of EGFR-tEV-treated mice (Figure 4D,E), indicating that EGFR-tEVs preferentially localize to tumor tissue and possess strong tumor-targeting potential in vivo.

### 2.5. EGFR-tEVs Enhanced Antitumor Effects in Colon Cancer In Vivo

**Figure 5:**
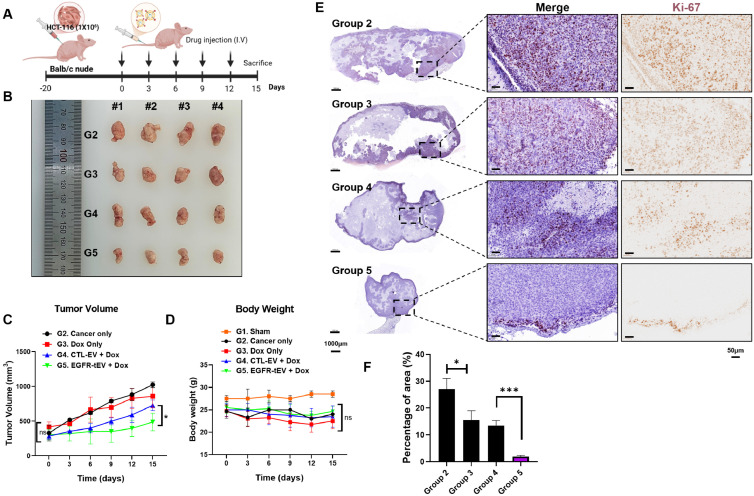
EGFR-tEVs enhance antitumor efficacy in an HCT-116 xenograft mouse model. (**A**) Schematic of the treatment protocol. HCT-116 cells were subcutaneously implanted into the left flank of BALB/c nude mice. After tumor establishment, treatments were administered via tail vein injection every 2–3 days for a total of five injections. Mice were sacrificed 15 days after the first injection. (**B**) Representative images of excised tumors from each group at the endpoint (n = 4 per group). (**C**) Tumor volume measurements over the treatment period. Tumor growth was significantly reduced in the EGFR-tEVs + Dox group compared with the CTL-EVs + Dox group at day 15. (**D**) Body weight changes are monitored during treatment. No statistically significant differences were observed among tumor-bearing treatment groups. Data are shown as mean ± SD (n = 4 per group). Statistical analysis was performed using one-way ANOVA with Tukey’s post hoc test. (**E**) Representative images of Ki-67 immunohistochemical staining in tumor sections (4× and 12.6× magnification). Scale bars = 1000 μm and 50 μm. (**F**) Quantification of Ki-67-positive areas analyzed using ImageJ (n = 3 tumors per group). Data are presented as mean ± SD. * *p* < 0.05, *** *p* < 0.001 ns, not significant.

HCT-116 cells were subcutaneously implanted into the left flank of Balb/c nude mice and allowed to grow for 20 days to establish solid tumors. The animals were divided into five groups as follows: G1, sham; G2, cancer cell only; G3, doxorubicin (Dox) alone; G4, CTL-tEVs + Dox; and G5, EGFR-tEVs + Dox. After tumor establishment, the indicated treatments were administered via tail vein injection five times at intervals of 2–3 days. Mice were sacrificed 15 days after the first injection, and tumors were harvested for analysis (Figure 5A). Comparison of tumor sizes among the groups revealed that the EGFR-tEVs + Dox-treated group (G5) exhibited the most pronounced inhibition of tumor growth. On day 15, tumor volumes were 789.8 ± 191.9 mm^3^ in G3 (Dox alone), 728.4 ± 66.2 mm^3^ in G4 (CTL-tEVs + Dox), and 487.5 ± 108.8 mm^3^ in G5 (EGFR-tEVs + Dox) (Figure 5B,C). Compared with G3, G4 showed a modest 7.8% reduction in tumor volume, which was not statistically significant. In contrast, G5 demonstrated a significant 38.3% reduction compared with G3. Furthermore, tumor volume in G5 was significantly reduced by 33.1% compared with G4, indicating that EGFR-targeted tEVs markedly enhanced the antitumor efficacy (Figure 5C). To evaluate systemic toxicity, body weight changes were monitored throughout the experimental period. All tumor-bearing groups (G2–G5) exhibited a general trend of body weight loss compared with the sham group (G1). However, no statistically significant differences in body weight were observed among the treatment groups (G2–G5) (Figure 5D), suggesting that EGFR-tEV-mediated drug delivery did not induce additional systemic toxicity.

To assess the anti-proliferative effect, immunohistochemical staining for Ki-67, a well-established marker of cell proliferation, was performed [26]. Representative images at 4× and 12.6× magnification showed nuclear Ki-67-positive staining in tumor tissues (Figure 5E). Quantitative analysis revealed that G5 exhibited a markedly lower percentage of Ki-67-positive area compared with G3 and G4 (Figure 5F). Specifically, G3 showed a significant 42.6% reduction compared with the cancer-only group (G2), while G4 and G5 demonstrated significantly greater reductions, with G5 showing the lowest Ki-67 expression among all tumor-bearing groups. Notably, G5 showed the most potent inhibitory effect, with the Ki-67-positive area decreasing from 10.98% in G4 to 1.88%, representing a remarkable 82.8% reduction in the proliferation index.

### 2.6. In Vivo Toxicity Evaluation of EGFR-tEVs

To evaluate the in vivo toxicity of EGFR-tEVs, major organs, including the liver, kidney, and spleen, were subjected to H&E staining. Histological examination revealed no observable pathological abnormalities in any treatment group. In the liver, hepatocytes maintained normal morphology without evidence of structural disruption. In the kidney, both glomeruli and renal tubules appeared intact, with no signs of inflammatory infiltration or tissue damage. Similarly, the spleen exhibited normal architecture without significant alterations in cell size or morphology (Supplementary Figure S1). These findings suggest that EGFR-tEV-based treatment did not induce histopathological toxicity in major organs. Additionally, blood samples were collected at the end of the experiment to assess systemic toxicity. Serum levels of sodium (Na^+^), potassium (K^+^), chloride (Cl^−^), C-reactive protein (CRP), alkaline phosphatase (ALP), and blood urea nitrogen (BUN) were measured. No significant differences were observed among the experimental groups, and all parameters remained within the normal physiological range (Supplementary Table S1). Collectively, these results demonstrate that EGFR-tEVs-mediated drug delivery does not cause detectable systemic or organ-specific toxicity in vivo.

## 3. Discussion

This study demonstrates that EGFR-targeted extracellular vesicles (EGFR-tEVs) enhance the therapeutic efficacy of doxorubicin in EGFR-overexpressing colorectal cancer models while maintaining a favorable safety profile. By engineering EVs to display the GE11 peptide, we aimed to improve tumor-specific delivery through receptor-mediated targeting. Our in vitro and in vivo findings consistently support the hypothesis that enhanced cellular internalization and tumor accumulation contribute to improved antitumor outcomes. The precision of our EGFR-targeted delivery system was further validated through comparative studies using the CCD-18co normal colon fibroblast cell line, which lacks significant EGFR expression. The observation that EGFR-tEVs exhibited minimal uptake and no significant apoptosis induction in these normal cells, in contrast to the potent effects seen in HCT-116 cancer cells, underscores the refined selectivity. This discrimination between cancerous and non-cancerous cells highlights the potential of EGFR-tEVs to circumvent the dose-limiting toxicities typically associated with free doxorubicin.

**Figure 6:**
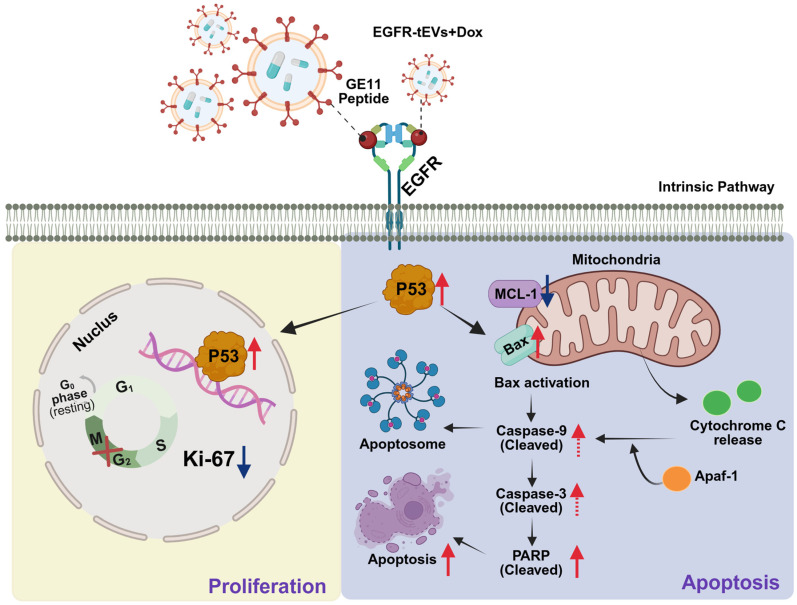
Proposed model of EGFR-tEVs-mediated enhancement of antitumor activity. Schematic representation of the proposed mechanism underlying EGFR-tEVs-mediated tumor suppression. Enhanced cellular uptake of EGFR-tEVs is associated with increased expression of pro-apoptotic markers (p53, BAX) and cleaved PARP1, along with reduced Ki-67 expression in vivo, indicating decreased tumor cell proliferation. Red and blue arrows denote increased and decreased expression, respectively. Solid arrows indicate experimentally observed changes, whereas dashed arrows represent proposed signaling pathways based on the previous literature. Created in BioRender. Chan mi, L. (2026) https://BioRender.com/qqr4fgs.

In EGFR-overexpressing HCT-116 cells, EGFR-tEVs showed significantly increased uptake compared with control EVs, indicating effective receptor-associated internalization. This enhanced uptake correlated with increased expression of pro-apoptotic markers, including p53, BAX, and cleaved PARP1. In vivo, EGFR-tEVs demonstrated preferential tumor accumulation and significantly reduced tumor volume and Ki-67 expression compared with both free doxorubicin and non-targeted EV groups. Collectively, these data suggest that EGFR-mediated targeting enhances functional drug delivery, leading to amplified apoptotic signaling and suppressed tumor cell proliferation. Although downstream apoptotic events such as cytochrome c release and caspase activation were not directly examined in this study, the proposed schematic summarizes the likely signaling pathway based on our findings and previous reports [27,28]. In this study, the increased expression of p53 and Bax, along with PARP1 cleavage, suggests the activation of the mitochondrial apoptotic pathway. However, we acknowledge that the direct measurement of intermediate events, such as cytochrome c release from the mitochondria to the cytosol and the subsequent activation of the caspase-9 and caspase-3 cascade, was not performed. While the p53-Bax-PARP1 axis is a well-established signaling pathway in drug-induced apoptosis [29], the absence of direct caspase activity assays remains a limitation. Future studies should incorporate these assays to definitively confirm the specific involvement of the mitochondrial-dependent caspase cascade in EGFR-tEV-mediated cell death (Figure 6). In contrast, while an increase in p53 expression was observed in normal colon fibroblasts (CCD18-co) following Dox treatment, there was no significant activation of Bax or cleavage of PARP1. Notably, no discernible differences in these apoptotic markers were found among the EGFR-tEVs, CTL-EVs, and Dox-only groups, suggesting that our delivery system does not induce additional cytotoxicity in non-cancerous cells. This lack of apoptotic response in normal cells, despite p53 induction, may indicate that p53-mediated survival pathways—such as cell cycle arrest or DNA repair—remain predominant over pro-apoptotic signaling in a non-malignant context [30,31]. Recent studies highlight that the functional outcome of p53 activation is highly context-dependent, where normal cells often prioritize genomic integrity maintenance over immediate programmed cell death under moderate stress [32]. Collectively, these data suggest that the EGFR-tEVs system minimizes off-target drug exposure and toxicity in normal cells while effectively amplifying apoptotic signaling specifically within the targeted cancer cells.

Despite the promising results, this study has several limitations that warrant further discussion. First, while EGFR-tEVs exhibited enhanced tumor accumulation, a significant portion was still sequestered by the reticuloendothelial system (RES), particularly in the liver and spleen, as observed in our IVIS data. This remains a common hurdle for EV-based therapies, as rapid systemic clearance can limit the fraction of the dose reaching the target tumor site. Second, the encapsulation efficiency (EE%) of doxorubicin was approximately 10–13%. Although this was sufficient to achieve a potent therapeutic effect due to targeted internalization, it remains lower than that of synthetic liposomes. To further improve the therapeutic window, future studies will focus on optimizing loading techniques—such as electroporation, sonication, or pH-gradient methods [33,34]—to maximize the drug-to-protein ratio in EV-based delivery systems. Furthermore, the quantification of drug loading capacity (LC%) in this study was based on the total protein mass determined by the BCA assay. Although we followed standard purification protocols to isolate EVs, the total protein mass may include co-isolated non-EV proteins, which could potentially affect the precision of the LC% calculation. Additionally, our current methodology does not distinguish between doxorubicin encapsulated within the EV lumen and that adsorbed onto the EV surface. This distinction is critical as it can influence drug release kinetics and overall therapeutic efficacy. While we used standard centrifugation and washing steps to remove unencapsulated drugs, the possibility of surface adsorption remains. Therefore, further investigations employing protease protection assays to distinguish encapsulated cargo or high-performance liquid chromatography (HPLC) for more precise quantification and release kinetic profiles are warranted to optimize the EGFR-tEV delivery system [35,36,37]. Another consideration is the heterogeneity of EGFR expression among colorectal cancer patients. Since EGFR-targeted delivery depends on receptor availability, patient stratification based on EGFR expression levels may be critical for maximizing therapeutic benefit [38]. Future investigations using patient-derived xenografts and immunocompetent models will provide further insight into the clinical applicability of this strategy. Lastly, while our study demonstrates that EGFR-tEVs + Dox significantly modulates universal apoptotic markers such as p53, Bax, and PARP1, evaluating colorectal cancer (CRC)-specific protein markers could provide further insight into the tissue-specific therapeutic mechanisms. Markers such as Carcinoembryonic Antigen (CEA) or Cyclooxygenase-2 (COX-2) are often implicated in CRC progression and chemoresistance [39,40]. Although not examined in this study, the impact of our targeted delivery system on these CRC-specific pathways warrants further investigation in future studies to fully elucidate its translational potential in clinical CRC therapy.

Compared with conventional EGFR-targeted therapies such as Cetuximab, which may be limited by acquired resistance or immune-related adverse effects, EV-based targeting offers potential advantages, including intrinsic biocompatibility and low immunogenicity. Furthermore, unlike antibody–drug conjugates that rely on chemical conjugation strategies, EVs provide a biologically derived lipid bilayer system capable of interacting with hydrophobic compounds while facilitating cellular uptake through membrane fusion and endocytosis pathways [10,41]. The significant enhancement in antitumor efficacy achieved through EGFR-targeting highlights the feasibility of improving drug delivery efficiency while maintaining an excellent safety profile. Our findings demonstrate that by utilizing GE11-mediated surface engineering, it is possible to achieve robust tumor suppression and a drastic reduction in cell proliferation markers, such as Ki-67, without the need for increasing systemic drug dosages. This approach underscores the potential of EGFR-tEVs to optimize the therapeutic window of doxorubicin, ensuring effective drug activity within malignant tissues while sparing normal cells from off-target toxicity.

Finally, the modular nature of this platform enables potential adaptation for the delivery of other therapeutic cargos, including nucleic acids or gene-editing systems [42,43]. From a translational perspective, the use of HEK293T cells—an industry-standard cell line—ensures that our EGFR-tEV platform possesses high scalability for biopharmaceutical manufacturing. The production process is fundamentally compatible with established large-scale purification technologies, such as tangential flow filtration (TFF), which can be integrated in future clinical-grade production to ensure consistency and purity. This manufacturability, combined with the superior intracellular delivery compared to conventional monoclonal antibodies, highlights the potential of EGFR-tEVs as a viable next-generation therapeutic strategy for CRC. Optimization of drug loading efficiency, scalable EV production, and rigorous characterization in accordance with established EV research guidelines will be necessary steps toward clinical translation.

## 4. Materials and Methods

### 4.1. Cell Culture

HEK293T cells were cultured in DMEM medium (High glucose, HyClone, South Logan, UT, USA). HCT-116 and CCD-18Co cells were cultured in RPMI-1640 medium (HyClone, South Logan, USA) supplemented with 10% FBS (Fetal Bovine Serum) (Gibco, Grand Island, NY, USA) and HEPES (Welgene, Gyeongsangbuk-do, Republic of Korea). All cell lines were obtained from the Korean Cell Line Bank (Jongno-gu, Seoul, Republic of Korea) and routinely tested for mycoplasma contamination. The cells were maintained in a humidified incubator at 37 °C with 5% CO_2_.

### 4.2. Plasmid DNA Cloning

The GE11 peptide (YHWYGYT-PQNVI, dodecapeptide), which targets EGFR, was synthesized and introduced into *E. coli* following the bacterial transformation protocol. To select the transformed cells, colonies were grown on LB agar plates (LPS solution, Maryville, TN, USA) containing 0.1 mg/mL ampicillin (Welgene, Gyeongsan-si, Republic of Korea) and subsequently transferred to LB medium (LPS solution, LB-05, USA) for further growth. The plasmid DNA was extracted using the Midi Prep Kit (Macherey-Nagel, North Rhine-Westphalia, Germany) and quantified using the NanoDrop spectrophotometer (Thermo Fisher, ND-2000, Wilmington, DE, USA).

HEK293T cells were cultured at a density of 5 × 10^6^ cells per plate in 100 mm dishes. After 24 h of incubation, the medium was replaced with 5 mL of serum-free Opti-MEM (Gibco, Waltham, MA, USA). Plasmid DNA and Lipofectamine (Invitrogen, Carlsbad, CA, USA) were added at a ratio of 1 µg plasmid DNA to 2 µL Lipofectamine. The mixture was incubated for 5 min at room temperature (RT). Target plasmid DNA (4 µg) was then added, and the mixture was incubated for an additional 15 min at RT. This mixture was added to the cells and incubated for 6 h. The medium was then replaced with DMEM/High glucose medium containing 10% FBS and 1% P/S. After 12 h, the medium was replaced with MEM without any additives, and the cells were cultured for another 24 h. The conditioned medium (CM) was collected the following day.

### 4.3. Extracellular Vesicles (EVs) Extraction

Conditioned media (CM) were collected from cultured cells and centrifuged at 2000× *g* for 30 min at 4 °C to remove cells and debris. The resulting supernatant was transferred to a clean ultracentrifuge tube and subjected to ultracentrifugation at 100,000× *g* for 70 min at 4 °C. The EV-containing pellet was resuspended in Dulbecco’s Phosphate-Buffered Saline (DPBS, Welgene, Gyeongsan, Republic of Korea) to wash away contaminating proteins and other soluble components, and ultracentrifuged again at 100,000× *g* for 70 min at 4 °C. The final EV pellet was resuspended in DPBS for subsequent experiments. All steps were performed at 4 °C to preserve EV integrity.

### 4.4. Nanoparticle Tracking Analysis (NTA)

The EVs, diluted 1/10, were injected into the ExoCopeTM device (Exosome Plus, Seoul, Republic of Korea), and the flow was stabilized to track the Brownian motion of the particles. The particles were monitored using the automatic tracking mode, and the size distribution was calculated. The results included both the size distribution and concentration distribution, and a graph visually representing the nanoparticle size distribution was generated.

### 4.5. Transmission Electron Microscopy (TEM)

Chemical fixation was performed prior to imaging to preserve the structural integrity of the isolated EVs. Transmission electron microscopy was conducted using a Hitachi HT7800 microscope (Hitachi, Tokyo, Japan) with technical assistance from a trained operator at The Catholic University. TEM enabled high-resolution imaging at the nanometer scale, allowing for detailed morphological characterization and size analysis of the EVs.

### 4.6. Cellular Uptake Assay

Cells were seeded at a density of 5 × 10^5^ cells per well in 8-well chamber slides (SPL, Gyeonggi, Republic of Korea) and cultured for 24 h prior to treatment. Extracellular vesicles (EVs) were labeled with 1 μM Dil; DilC18(3) (Invitrogen, CA, USA), incubated at 37 °C for 20 min in the dark, and unbound dye was removed by ultracentrifugation followed by concentration using Amicon Ultra-4 centrifugal filters (100 kDa MWCO; Millipore, Burlington, MA, USA). EV concentration was determined by nanoparticle tracking analysis and normalized to 1 × 10^7^ particles/mL. Cells were incubated with Dil-labeled EVs (1 × 10^7^ particles/mL) at 37 °C for 24 h. Additional controls included a Dil-only treatment group and an unlabeled EV treatment group. After incubation, cells were washed three times with PBS and fixed with 4% paraformaldehyde (Biosesang, Gyeonggi, Republic of Korea) for 10 min at room temperature. Nuclei were counterstained with DAPI-containing antifade mounting medium (Vector Laboratories, CA, USA). Images were acquired using a confocal laser scanning microscope (LSM700; ZEISS, Oberkochen, Germany), and fluorescence intensity per cell was quantified using ImageJ software (NIH, USA) from at least three random fields per sample (≥10 cells).

### 4.7. Drug Loading and Efficiency Analysis

To load the cargo, EVs (1 × 10^7^ particles/mL) were mixed with 2 μM doxorubicin and incubated at 37 °C for 2 h. Following incubation, the mixture was processed through an Amicon Ultra 0.5 centrifugal filter (Millipore, Burlington, MA, USA) to eliminate any unbound drug. The filter was equilibrated with 500 μL PBS (14,000× *g*, 5 min) before use. The sEV-doxorubicin complex was collected by centrifuging at 1000× *g* for 10 min, then recovered by filter inversion into a clean collection tube. Encapsulated doxorubicin was liberated using 1% Triton X-100 for 20 min at room temperature. Quantification was performed via fluorescence spectroscopy (Ex: 480 nm, Em: 590 nm) using a BioTek microplate reader (BioTek Instruments, Winooski, VT, USA), referencing a standard curve (0–32 μM). The encapsulation efficiency (EE%) and loading capacity (LC%) were determined using the following formulas:

- EE (%) = (Mass of doxorubicin in EVs/Initial mass of doxorubicin added) × 100
- LC (%) = (Mass of doxorubicin in EVs/Total EV protein mass) × 100

Total protein was assessed via BCA protein assay (Thermo Scientific), with all measurements conducted in triplicate.

### 4.8. In Vitro Assay (Co-Incubation of EVs and Doxorubicin)

For Western blot and qRT-PCR analyses, cells were seeded at a density of 5 × 10^5^ cells per well in 6-well plates and cultured for 24 h prior to treatment. Treatment samples were prepared with a total volume of 100 μL, containing EVs at 1 × 10^7^ particles/mL and doxorubicin at 2 μM, and the remaining volume was adjusted with DPBS. The mixture was incubated at 37 °C for 2 h and then applied to each well. After 24 h of incubation, cells were collected for downstream analyses.

### 4.9. Western Blot

Cell lysates were prepared following the drug treatment. The cells were washed with PBS and then lysed in RIPA Lysis buffer (ATTO Corporation, Tokyo, Japan) supplemented with protease inhibitors and phosphatase inhibitors. Protein concentrations were measured using the BCA protein assay kit (Thermo Scientific, Rockford, IL, USA). Equal amounts of protein were loaded onto an SDS-PAGE gel and separated by electrophoresis at 100 V for 2 h in Tris-glycine SDS running buffer. After electrophoresis, the proteins were transferred to a nitrocellulose membrane (Cytiva, Freiburg, Germany) at 100 V for 2 h. The membrane was blocked with Block solution (Translab, Seoul, Republic of Korea) for 1 h, and incubated overnight at 4 °C with the primary antibody at 1:500 or 1:1000 dilution. HSP70 (Cat#. 4876), CD9 (Cat#. 13403), CD81 (Cat#. 56039), EGFR (Cat#. 4267), BAX (Cat#. 5023), and PARP1 (Cat#. 9542) from Cell Signaling Technology (Danvers, MA, USA); and Cytochrome C (Cat#. sc-13156), p53 (Cat#. sc-126), and GAPDH (Cat#. sc-365062) from Santa Cruz Biotechnology (Dallas, TX, USA). After three washes with TBST, the membranes were incubated with HRP-conjugated secondary antibodies at 1:2000 dilution for 2 h at 36 °C: Goat anti-mouse IgG F(ab’)_2_ (Cat#. ADI-SAB-100 J, Enzo Life Sciences, Farmingdale, NY, USA) or WestVision™ Peroxidase Polymer Anti-Rabbit IgG (Cat#. WB-1000, Vector Laboratories, Burlingame, CA, USA). Protein bands on the membrane were detected using a Western blot Hyper HRP substrate (TAKARA, Mountain View, CA, USA) and imaged with a visible fluorescent western imaging system (Azure Biosystems, Dublin, CA, USA). Densitometric analysis of the protein bands was performed using ImageJ software (National Institutes of Health, Bethesda, MD, USA). The intensity of the target protein bands was normalized to the intensity of housekeeping proteins such as GAPDH.

### 4.10. Quantitative Real-Time Poly Chain Reaction (qRT-PCR)

Total RNA was extracted using TRIzol Reagent (Ambion, Carlsbad, CA, USA). RNA concentration and quality were assessed using a NanoDrop spectrophotometer (Thermo Fisher Scientific, Waltham, MA, USA), and only samples with an A260/A280 ratio between 1.8 and 2.0 were used. cDNA synthesis was performed with 0.5 µg of total RNA using the ReverTra Ace qPCR RT Master Mix with gDNA Remover (TOYOBO, Kita-Ku, Japan) in a 20 µL reaction volume on a Thermal Cycler Dice Touch (TAKARA, Sydney, Australia). Quantitative PCR was performed using SYBR qPCR Mix (TOYOBO, Osaka, Japan) on a Real-Time StepOnePlus System (Thermo Fisher Scientific, Waltham, MA, USA). The reaction mixture (20 µL) contained 10 µL of SYBR qPCR Mix, 1 pM of forward and reverse primers, and 1 µL of cDNA template. The PCR cycling conditions were as follows: 1 min at 95 °C, followed by 40 cycles of 15 s at 95 °C and 45 s at 60 °C. Melt curve analysis was performed to verify the amplification specificity. Relative gene expression was calculated using the ΔΔCt method, with GAPDH and β-actin as internal controls. The primer sequences are listed in Supplementary Table S2.

### 4.11. Animal Experimentation

All animal experiments were approved by the Institutional Animal Care and Use Committee (IACUC) of the College of Medicine, The Catholic University of Korea (Approval number: 2024-0211-02) and conducted in accordance with the ARRIVE guidelines. All animals underwent a 7-day acclimatization period prior to the start of the experiments. Mice were housed in a specific pathogen-free facility under controlled environmental conditions and provided with food and water ad libitum. All procedures were performed under isoflurane anesthesia, and humane endpoints were applied to minimize animal suffering. Blood samples were collected via cardiac puncture under anesthesia. CO_2_ inhalation was used for euthanasia.

### 4.12. Xenograft Mouse Model

HCT-116 cells (1 × 10^6^ cells in 100 µL) were injected into the left flank of Balb/c nude mice (6 weeks old, approximately 20 g). Drug treatment began when the tumor volume reached 304.10 mm^3^ ± 104.98 mm^3^. All treatment groups received 2 mg/mL doxorubicin co-incubated in 1 × 10^7^ P/mL EVs, with a total injection volume of 120 µL. Injections were administered three times a week, for a total of five doses. Mice were sacrificed on the 15th day following the first injection. Prior to drug administration, the mice were measured for their long and short axes using a caliper, and their body weight was recorded. Animal monitoring was conducted according to the scoring system outlined in the Humane Endpoints in Animal Experiments for Biomedical Research, and experiments were humanely terminated according to these criteria. In cases where colorectal cancer progression resulted in >20% body weight loss due to reduced food and water intake or tumor size exceeded 10% of body weight, animals were euthanized using CO_2_ inhalation regardless of the standard endpoints.

### 4.13. In Vivo Imaging System (IVIS)

A noninvasive imaging system was used to monitor EV distribution and dynamic movement in vivo. After establishing a xenograft mouse model, EVs were extracted at a concentration of 1 × 10^7^ P/mL and labeled with the fluorescent dye DiR, DiIC18(7) (1,1′-Dioctadecyl-3,3,3′,3′-Tetramethylindotricarbocyanine Iodide). The labeled EVs were intravenously (IV) injected into the mice. At 24 and 48 h post-injection, the tissue-specific distribution of EVs was imaged using the IVIS (Optical In Vivo Imaging System-IVIS Lumina XRMS, PerkinElmer, Hopkinton, MA, USA).

### 4.14. Immunohistochemistry

Tissue blocks from each group were sectioned, mounted on slides, and dried. The slides were then incubated in a 60 °C dry oven for 1 h. Slides were deparaffinized and washed for 5 min in running water. Antigen retrieval was induced by immersing the slides in 1× TE buffer (pH 9.0) and heating for at least 30 min, followed by another 5 min of washing in running water. Tissue borders were outlined with a Dako pen, and distilled water was applied to prevent the tissue from drying. After removing the distilled water, H_2_O_2_ solution was applied, and the slides were incubated for 3 min. The slides were then washed with 1× PBS, followed by the application of a blocking agent, which was incubated for 30 min. After PBS washing, the slides were incubated overnight at 4 °C with the primary antibody (Anti-Ki67 antibody, 1:1000, Abcam, Cambridge, UK) in a humidified chamber. The next day, following PBS washing, the slides were incubated with the secondary antibody for 30 min. After another wash, the VECTAIN Elite ABC kit (Vector, Newark, CA, USA) was applied, and the slides were incubated for 30 min before being washed again. Subsequently, the substrate working solution (ImmPACT NovaRED Substrate Kit, Vector, CA, USA) was applied, and the coloration was monitored and adjusted accordingly. Finally, the slides were stained with Mayer’s hematoxylin for 15 s to complete the staining process.

### 4.15. Statistical Analyses

All statistical analyses were performed using GraphPad Prism version 8.0.2. Normality of the data was assessed using the Shapiro–Wilk test. For normally distributed data, parametric tests were used, including Student’s *t*-test for two-group comparison, and one-way ANOVA followed by Tukey’s post hoc test for multiple groups comparison. For data involving two independent variables, such as tumor volume and body weight changes over time, two-way ANOVA followed by Sidak’s or Bonferroni’s post hoc test was employed. For non-normally distributed data, nonparametric tests (e.g., Mann–Whitney U test or Kruskal–Wallis test) were applied. All data are presented as mean ± standard deviation (SD). The level of statistical significance was set at * *p* < 0.05, ** *p* < 0.01, and *** *p* < 0.001.

## 5. Conclusions

This study provides evidence that EGFR-targeted extracellular vesicles (EGFR-tEVs) can modulate apoptosis-related signaling and suppress tumor cell proliferation in EGFR-expressing colorectal cancer. Treatment with EGFR-tEVs in combination with doxorubicin resulted in significant tumor growth inhibition while maintaining minimal systemic toxicity. In vitro and in vivo analyses confirmed increased p53 and Bax expression, elevated cleaved PARP1, and reduced Ki-67 expression, supporting the involvement of apoptosis and proliferation pathways in the observed antitumor effects.

## Data Availability

The datasets generated or analyzed during the current study are available from the corresponding author upon reasonable request.

## Supplementary Materials

The following supporting information can be downloaded at: https://www.mdpi.com/article/10.3390/ijms27083693/s1.
