# Supplementary material for: EGFR-Targeted Extracellular Vesicles Potentiate Doxorubicin-Induced Apoptosis and Tumor Suppression in Colorectal Cancer
Source: Int J Mol Sci. 2026 Apr 21;27(8):3693. doi: 10.3390/ijms27083693 (PMC13116487; doi:10.3390/ijms27083693)

# EGFR-Targeted Extracellular Vesicles Potentiate Doxorubicin-Induced Apoptosis and Tumor Suppression in Colorectal Cancer

**Chan Mi Lee [1,2], Ji Won Choi [1], Do Sang Lee [1,2], Joo Won Moon [1], Jin Beom Cho [1,3] and Jung Hoon Bae [1,2,*]**

[1] Central Institute of Surgery, Seoul St. Mary's Hospital, College of Medicine, The Catholic University of Korea, Seoul 06591, Republic of Korea; cksal7873@cmcnu.or.kr (C.M.L.); qrt97@cmcnu.or.kr (J.W.C.); dosangs@catholic.ac.kr (D.S.L.); mey8010@cmcnu.or.kr (J.W.M.); jinbum21@catholic.ac.kr (J.B.C.)

[2] Department of Surgery, Seoul St. Mary's Hospital, College of Medicine, The Catholic University of Korea, Seoul 06591, Republic of Korea

[3] Division of Colorectal and Anorectal Surgery, Department of Surgery, Uijeongbu St. Mary's Hospital, College of Medicine, The Catholic University of Korea, Uijeongbu 11765, Republic of Korea

* Correspondence: 21000592@cmcnu.or.kr

## Supplementary material and method

### Hematoxylin and eosin staining of tissue and tumor sections

Tumor and tissue samples were paraffin-embedded and sectioned. Sections were deparaffinized using eco-friendly solutions, followed by sequential dehydration through graded ethanol (100% twice and 75%) and rinsing in tap water. After pre-treatment, sections were stained with hematoxylin for 3–5 min, differentiated, blued, and rinsed thoroughly. Eosin staining was performed in 95% ethanol for 15 s. Sections were then dehydrated through absolute ethanol (three changes), n-butanol (two changes), and xylene (two changes), mounted with coverslips, and examined under a fluorescence microscope. To analyze the histological features, the slides were scanned with a Pannoramic SCAN II system (3DHISTECH Ltd, Hungary). Representative images were captured as for analysis.

### Blood serum toxicity test

The mouse blood was collected via cardiac puncture, and the plasma and serum were separated by centrifugation. Only the plasma was used for analysis. Blood analysis, including liver function (ALT), kidney function (BUN), and electrolyte levels, was conducted using the automated analyzer FUJI DRI-CHEM NX500 (FUJIFILM Corporation, Tokyo, Japan). Results were provided as digital outputs and presented in quantitative values.

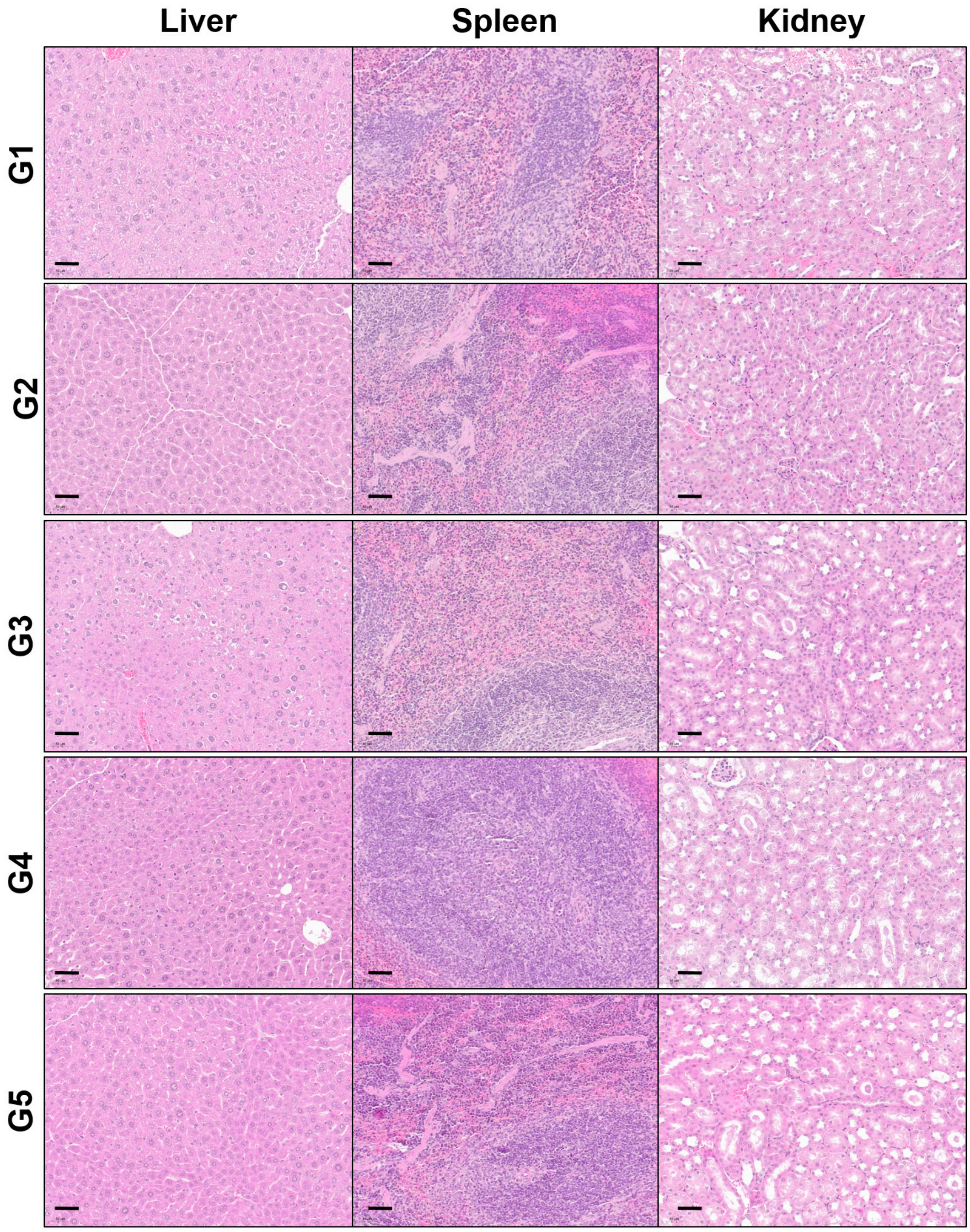


**Supplementary Figure S1. Toxicity assessment in liver, kidney, and spleen tissues.**

H&E staining showed preserved cellular arrangements and tissue structures across all groups. The liver exhibited uniformly stained hepatocytes and cytoplasm without abnormalities. Kidney tubules and glomeruli maintained normal morphology with no inflammation. The spleen showed no significant changes in cell size or shape. Scale bar = 50μm.

| | Normal Range | Group 1 | Group 2 | Group 3 | Group 4 | Group 5 |
|---|---|---|---|---|---|---|
| **$Na^+$, mEq/l** | 140 - 150 | 146 | 149 | 148 | 144 | **152** |
| **$K^-$, mEq/l** | 3.5 - 5.5 | 5.2 | 4.1 | 4.9 | 4.2 | 4.9 |
| **$Cl^-$, mEq/l** | 100 - 115 | 108 | 110 | 109 | 105 | 113 |
| **Na-K, mEq/l** | 30 - 40 | 28.1 | 36.3 | 30.2 | 34.3 | 31 |
| **CRP, mg/dl** | 0 - 0.5 | 0.3 | 0.3 | 0.3 | 0.3 | 0.3 |
| **ALP, U/l** | 30 - 200 | 377 | 243 | 252 | 256 | 261 |
| **BUN, mg/dl** | 10 - 30 | 18.5 | 21.2 | 25 | 18.8 | 29.7 |

**Supplementary Table S1. Blood toxicity evaluation**

blood samples collected after the experiment were analyzed for $Na^+$, $K^-$, $Cl^-$, Na–K, CRP, ALP, and BUN levels. All parameters remained within normal ranges across all groups, confirming the absence of systemic toxicity.

| Gene Bank Accession no. | Organism | Primer | | Primer sequence (5’→3’) |
|---|---|---|---|---|
| NM_001346900.2 | Homo sapiens | epidermal growth factor receptor (EGFR) | F | CAG CGC TAC CTT GTC ATT CA |
| | | | R | TGC ACT CAG AGA GCT CAG GA |
| NM_021960.5 | Homo sapiens | MCL1 apoptosis regulator, BCL2 family member (MCL1) | F | GAG GAG GAG GAG GAC GAG TT |
| | | | R | ACA TTC CTG ATG CCA CCT TC |
| NM_004324.4 | Homo sapiens | BCL2 associated X, apoptosis regulator (BAX) | F | TCT GAC GGC AAC TTC AAC TG |
| | | | R | TTG AGG AGT CTC ACC CAA CC |
| NM_001357943.2 | Homo sapiens | GAPDH | F | GAG TCA ACG GAT TTG GTC GT |
| | | | R | TTG ATT TTG GAG GGA TCT CG |

**Supplementary Table S2. Primer sequence**

# Western blot. RAW data

## Figure 1C

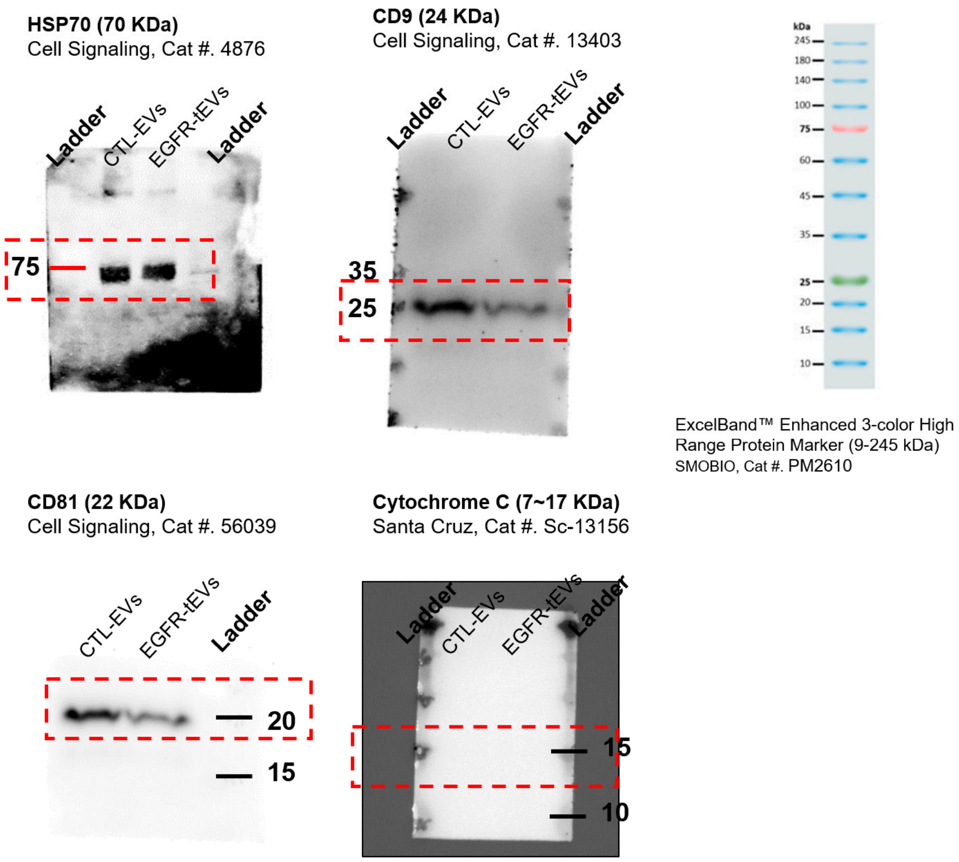


## Figure 2A

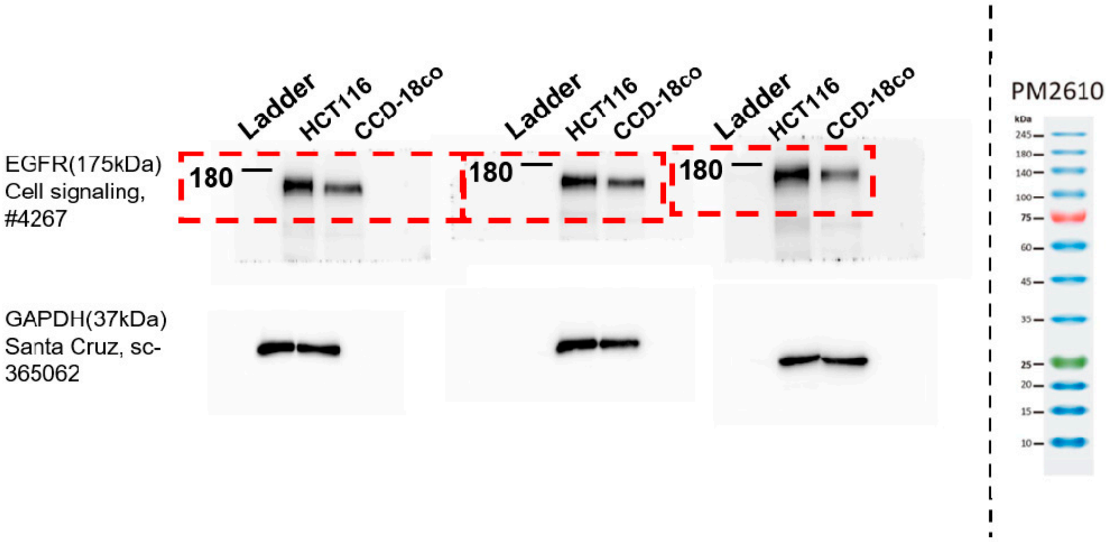

**Figure 3D**

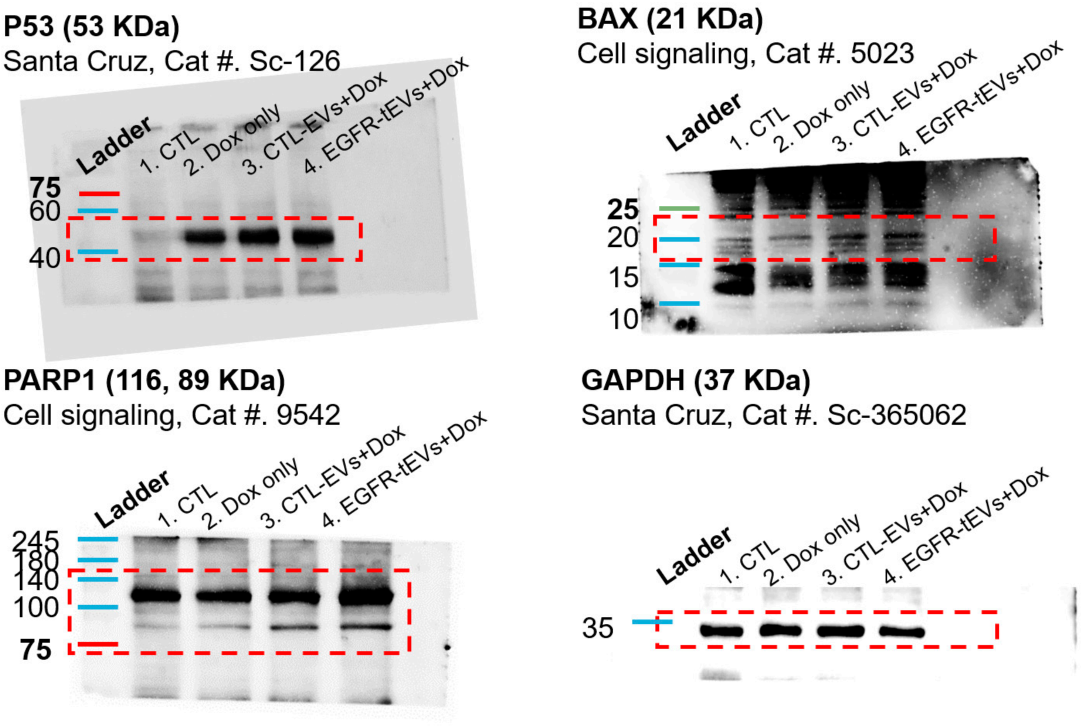


**Figure 3E**

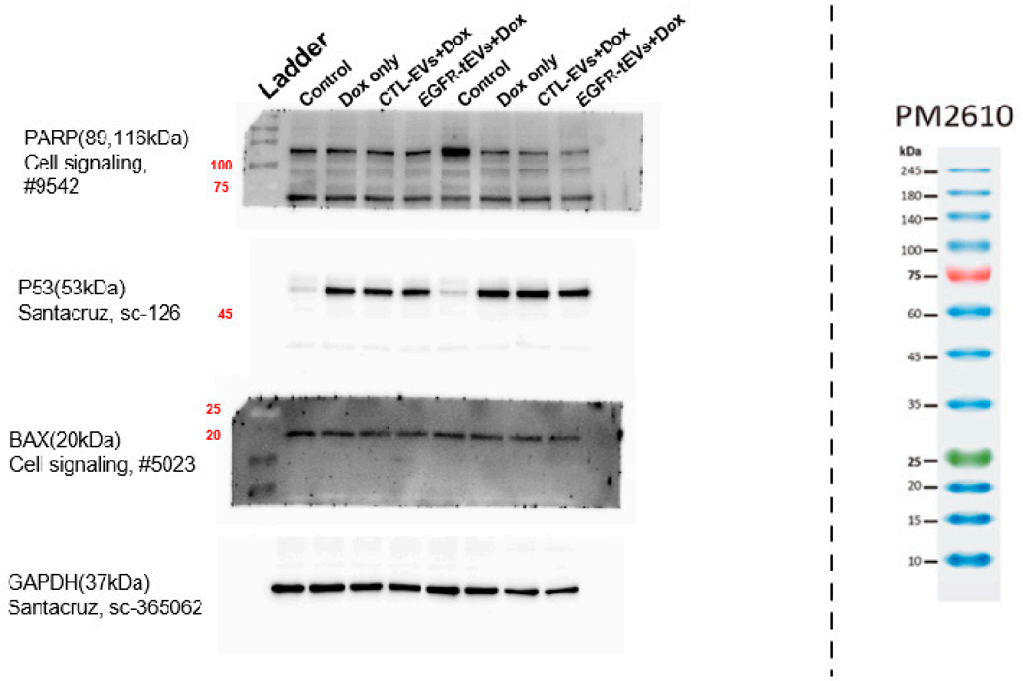

Supplement: Supplementary file 1 [file ijms-27-03693-s001.zip › Supplementary Figure File_JHB.pdf]
